# Identification of Potential Type II Diabetes in a Chinese Population with a Sensitive Decision Tree Approach

**DOI:** 10.1155/2019/4248218

**Published:** 2019-01-22

**Authors:** Dongmei Pei, Chengpu Zhang, Yu Quan, Qiyong Guo

**Affiliations:** ^1^Department of Family Medicine, Shengjing Hospital, China Medical University, Shenyang, Liaoning, China; ^2^Department of Informatics, Shengjing Hospital, China Medical University, Shenyang, Liaoning, China; ^3^Department of Radiology, Shengjing Hospital, China Medical University, Shenyang, Liaoning, China

## Abstract

**Background:**

Diabetes mellitus is a chronic disease with a steadfast increase in prevalence. Due to the chronic course of the disease combining with devastating complications, this disorder could easily carry a financial burden. The early diagnosis of diabetes remains as one of the major challenges medical providers are facing, and the satisfactory screening tools or methods are still required, especially a population- or community-based tool.

**Methods:**

This is a retrospective cross-sectional study involving 15,323 subjects who underwent the annual check-up in the Department of Family Medicine of Shengjing Hospital of China Medical University from January 2017 to June 2017. With a strict data filtration, 10,436 records from the eligible participants were utilized to develop a prediction model using the J48 decision tree algorithm. Nine variables, including age, gender, body mass index (BMI), hypertension, history of cardiovascular disease or stroke, family history of diabetes, physical activity, work-related stress, and salty food preference, were considered.

**Results:**

The accuracy, precision, recall, and area under the receiver operating characteristic curve (AUC) value for identifying potential diabetes were 94.2%, 94.0%, 94.2%, and 94.8%, respectively. The structure of the decision tree shows that age is the most significant feature. The decision tree demonstrated that among those participants with age ≤ 49, 5497 participants (97%) of the individuals were identified as nondiabetic, while age > 49, 771 participants (50%) of the individuals were identified as nondiabetic. In the subgroup where people were 34 < age ≤ 49 and BMI ≥ 25, when with positive family history of diabetes, 89 (92%) out of 97 individuals were identified as diabetic and, when without family history of diabetes, 576 (58%) of the individuals were identified as nondiabetic. Work-related stress was identified as being associated with diabetes. In individuals with 34 < age ≤ 49 and BMI ≥ 25 and without family history of diabetes, 22 (51%) of the individuals with high work-related stress were identified as nondiabetic while 349 (88%) of the individuals with low or moderate work-related stress were identified as not having diabetes.

**Conclusions:**

We proposed a classifier based on a decision tree which used nine features of patients which are easily obtained and noninvasive as predictor variables to identify potential incidents of diabetes. The classifier indicates that a decision tree analysis can be successfully applied to screen diabetes, which will support clinical practitioners for rapid diabetes identification. The model provides a means to target the prevention of diabetes which could reduce the burden on the health system through effective case management.

## 1. Introduction

Diabetes mellitus is a chronic disease that affects a large portion of the population with a steadfast increase in prevalence of diabetes globally, and such a tendency is projected to continue to rise over time as the population grows and ages. As reported by the World Health Organization (WHO), the number of people with diabetes has risen from 108 million in 1980 to 422 million in 2014 and will place diabetes as the seventh leading cause of death in 2030 [1]. Due to the chronic course of the disease combining with devastating complications, this disorder could easily cost medical care tens of billions of dollars. In a very recent report, the direct and indirect economic costs of diabetes in the United States of America in 2017 were as high as 327 billion dollars [2]. People with diabetes could easily carry a financial burden of about $13,700 per year [3].

Although the pathological mechanisms of diabetes appear to be closely associated with either a reduced production of insulin by the beta cells in the pancreas or the failures in transporting circulating glucose into the tissues via glucose receptors, the hyperglycemia and the subsequent complications involving the multiple organs and systems [4] could be managed by a modality of means including diet control, lifestyle modifications, and the effective therapeutic interventions including insulin administration [4, 5]. However, due to its insidious development, the early diagnosis of diabetes still remains as one of the major challenges medical providers are facing, and the satisfactory screening tools or methods are still required, especially a population- or community-based tool [6].

In the last decade, by constructing predictive models, an attempt to identify the factors that are potentially associated with the development of diabetes through data mining techniques has been made with some promising results in predicting or even capturing diabetes at its early stage [4, 7–12]. Among these techniques, the decision tree technique was widely used in the medical field in making diagnostic approaches during clinical practice [4, 11, 13–15]. By creating a set of simple classification rules, this simple but sensitive decision tree approach offers its unique capability of establishing a prediction toward a disease by extracting meaningful information from a large dataset which is composed of many attributable factors [4, 14, 16]. Therefore, the objective of the present study was to employ a decision tree method as a support system by rapid and automated identification of individuals with potential diabetes.

## 2. Methods

### 2.1. Study Population

Subjects whose age was ≥18 and who underwent the annual health check-up in the Department of Family Medicine of Shengjing Hospital of China Medical University from January 2017 to June 2017 were enrolled in this retrospective cross-sectional study. Informed consent was obtained from all participants. The Ethics Committee of Shengjing Hospital of China Medical University approved this study (reference number: 2017PS42K). By examining the medical record of each participant enrolled, a series of 9 variables pertinent to the health states that a patient could have at a given time point of his or her life were identified and used later for constructing a decision tree as reported in previous studies [8, 11, 17–20], and these variables are listed in Table 1.

### 2.2. Selection of Variables in the Study Design

A series of nine input variables, which were widely used by the others in establishing diabetes prediction models [8, 11, 17–20], were obtained through either direct observations or laboratory tests of each participant. In detail, the demographic characteristics such as age and gender were analyzed for all the subjects; family history of diabetes was defined as any family member previously having been diagnosed with diabetes (Yes = 1, No = 0); history of cardiovascular disease or stroke was defined as one previously having been diagnosed as having coronary heart disease or stroke (Yes = 1, No = 0); physical activity referred to those that took at least more than 30 minutes of exercise for 3 days in a week (More = 1, Less = 0); work-related stress has three levels according to the participants' subjective impression (High = 2, Moderate = 1, Low = 0); and salty food preference was defined as a person who prefers salty food in daily life (Yes = 1, No = 0). Anthropometric information contain body mass index (BMI) and hypertension. BMI was calculated as weight in kilograms divided by the square of height in meters (kg/m^2^), and a BMI ≥ 25 was defined as overweight; hypertension was defined as systolic blood pressure ≥ 140 mmHg or diastolic blood pressure ≥ 90 mmHg and/or current use of antihypertensive drugs for blood pressure control.

Diabetes diagnoses were made as either prediabetes or diabetes based on the fasting plasma glucose with a cut-off value of ≥5.6 mmol/L [8, 11], and each medical report was then marked as either diabetes or nondiabetes accordingly.

### 2.3. Data Selection

A total of 15,323 records were initially examined and analyzed for potential construction of a decision tree approach. In order to meet the strict criteria for building data mining algorithms, the dataset in which certain variables were missing cannot be used since the decision tree approach will not work with missing data points [21]. In doing so, 28.6% of the total data (4348 out of 15,323 records) failed to meet the criteria for data selection and was thus excluded accordingly. As a result, BMI (*N* = 1139, 26.0%), family history of diabetes (*N* = 313, 7.1%), history of cardiovascular disease or stroke (*N* = 1035, 23.6%), physical activity (*N* = 517, 11.8%), work-related stress (*N* = 618, 14.1%), salty food preference (*N* = 762, 17.4%), and with a past history of diabetes (*N* = 503, 3.3%) were discarded, respectively. With such a strict data filtration, the remaining 10,436 records from the eligible participants were used for further analysis in this study.

### 2.4. Classification Algorithm for Decision Tree Analysis

The final database consisted of 10 variables which were divided into 9 input variables and one target variable. The target variable consists of two classes, the presence of diabetes or no diabetes. Classification of individuals as being at risk for diabetes or not was performed using the decision tree classifier J48 (C4.5 algorithm), a robust classifier which operates with numeric and nominal attributes and was implemented in WEKA 3.8.1 (Waikato Environment for Knowledge Analysis, University of Waikato, New Zealand) [22]. With this technique, a tree was constructed to model the classification process based on the best informative attribute of the algorithm. At each iteration, the attribute was selected with the maximum gain ratio as the splitting attribute.

Data mining methods often divide the dataset into two parts: a training dataset and a testing dataset. The prediction model is first constructed on the training dataset and then tested on the testing dataset [23]. Consistent with these methods, the final dataset, consisted of 10,436 records, was randomly categorized into two groups, the training and test groups. The training group comprised of 7305 cases (70% of the whole dataset). The remaining 3131 cases (30% of the whole dataset) were allocated as the test group for model validation.

In the decision tree, the first variable (root) is the most important factor and variables far away from the root are the next important factors in classifying the data [4]. All the variables in one path are considered predictors (IF part), and the class label of the leaf node is the expected outcome (THEN part). To avoid overfitting and maintain parsimony, the model generated by the tree may be pruned by removing the nonessential terminal branches based on defined algorithms without affecting the classification accuracy [4, 24].

### 2.5. Model Evaluation

We used a confusion matrix to determine the performance of the decision tree for diabetes. In this study “diabetes” was defined as a positive event and “nondiabetes” was defined as a negative event. The confusion matrix for two classes was used to extract true positives, true negatives, false positives, and false negatives.

Using Statistical Package for the Social Sciences (SPSS) version 20.0, a chi-square test was used to compare the categorical characteristics between the nondiabetes and diabetes groups. To measure the performance of the model, we used accuracy, precision, and recall. A receiver operating characteristic (ROC) graph is a technique for visualizing, organizing, and selecting classifiers based on their performance. The area under the ROC curve (AUC) of the classifier can be described as the probability of the classifier to rank a randomly selected positive case higher than a randomly selected negative case.

## 3. Results

In this study, the characteristics of the 10,436 records that were divided into two groups (diabetes and nondiabetes) are shown in Table 1. Chi-square analysis revealed there was a significant difference between two groups as indicated in the table. We found that 1570 persons were diabetic and 8866 were not.

**Figure 1:**
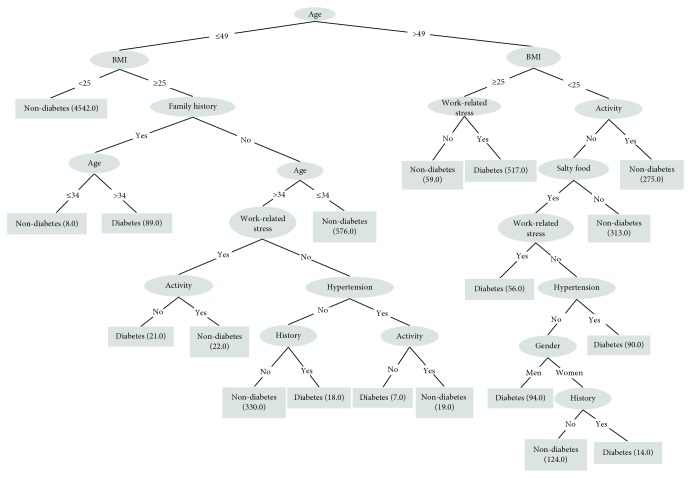
Decision tree approach with the training dataset in the model. The number in the bracket of each node represents the sample size.

A decision tree was built on the training dataset (*N* = 7305) while the testing dataset (*N* = 3131) was used to evaluate the model. By applying the J48 algorithm, a decision tree with 19 nodes and 20 leaves was built, and the results are illustrated in Figure 1. The tree shows that age is the most discriminatory attribute, followed by BMI, family history of diabetes, work-related stress, physical activity, salty food preference, hypertension, gender, and history of cardiovascular disease or stroke.  Table 2 lists all 19 IF-THEN rules created by the model.

The model was further evaluated for its accuracy by applying a confusion matrix analysis on the testing dataset, and the result is shown in Table 3. This prediction model had an accuracy of 94.2%; with such a high performance, 94.2% (2948 out of 3131 individuals) was correctly classified, whereas only 5.8% (183 out of 3131 individuals) was incorrectly classified. The area under the ROC curve (AUC) for the model was 94.8%, demonstrating that this model has achieved a higher accuracy in classifying the true positives rather than the false positives. Furthermore, the precision and recall values for the model were 94.0% and 94.2%, respectively, which were significantly balanced.

The decision tree demonstrated that among those participants with age ≤ 49, 5497 participants (97%) of the individuals were identified as nondiabetic, while age > 49, 771 participants (50%) of the individuals were identified as nondiabetic.

In the subgroup where people were 34 < age ≤ 49 and BMI ≥ 25, when with positive family history of diabetes, 89 (92%) out of 97 individuals were identified as diabetic, when without family history of diabetes, 576 (58%) of the individuals were identified as nondiabetic.

Work-related stress was identified as being associated with diabetes. In individuals with 34 < age ≤ 49 and BMI ≥ 25 and without family history of diabetes, 22 (51%) of the individuals with high work-related stress were identified as nondiabetic while 349 (88%) of the individuals with low or moderate work-related stress were identified as not having diabetes (Table 2).

## 4. Discussion

By having factored nine variables pertinent to the prediction of the development of diabetes in a community-based setting into a sensitive decision tree model, we systemically analyzed a large dataset solely collected from a Chinese ethnic population. Our decision tree approach yielded a highly accurate (94.2%) model with balanced precision (94.0%) and recall (94.2%), respectively. Although all nine variables such as age, gender, BMI, hypertension, history of cardiovascular disease or stroke, family history of diabetes, physical activity, work-related stress, and salty food preference carried certain critical values in making an early and noninvasive prediction of diabetes before the expensive laboratory tests become necessary, age plays a dominant role on the potential development of diabetes by serving as a single but the most discriminatory attribute to diabetes. Secondary to age, BMI also stood out as another critical predictive attribute. With BMI being greater than 25, the subjects with age older than 34, younger than or equal to 49, and a positive family history of diabetes tend to have a higher incidence in developing diabetes. Under such a circumstance, BMI itself could be considered a key predictor. In contrast, the work-related stress appeared to be an important predictive factor for those with age younger than 49. Our findings were consistent with those previous results [11, 25, 26]. As suggested in our decision tree model, the participants with BMI < 25 and younger than 49 carry a low risk of having diabetes. Similarly, as shown in rules 1 and 13 (Table 2), these subjects with BMI < 25 and active physical exercise tend to have a much less chance to develop diabetes. In addition to these findings, the work-related stress appeared to be another key risk factor as those aged people with a relatively higher BMI possess a relatively higher likelihood in their life to develop diabetes. Among several novel discoveries from our decision tree approach toward the prediction of diabetes in a community-based setting, combining multiple variables pertinent to the prediction of diabetes, medical care providers could take advantage of this simple but meaningful approach in their practice to identify those people who are at a high risk for diabetes [27]. It is thus that by screening candidates for potential diabetes, this decision tree approach becomes a powerful tool for the health care providers during their routine encounters with patients in a community-based setting. No doubt, a successful identification of a person with an early diabetes could not only reduce the chance for the patient to develop a full-blown diabetes with severe complications but also significantly mitigate the direct and indirect costs incurred. Additionally, the key features presented in the tree structure could further facilitate diabetes prevention through community interventions, as individuals who are at a high risk of developing diabetes could quickly and easily be identified and targeted for a variety of education programs specifically designed for diabetes. Indeed, several large-scale trials have demonstrated the benefits of preventing diabetes with simple lifestyle interventions [8, 11, 28–31]. Appropriately implemented preventive interventions in the early stage of the disease could render the progression of the disease to be less aggressive in its development as well as less expensive to both households with individuals with diabetes and the health care system. It is worth pointing out that such an approach can be extended to include other chronic disease managements in a community setting. Nevertheless, the present study could be improved if it could have been conducted in multiple sites. Indeed, a longitudinal approach with additional prospective data would better to explore the relationship among risk factors and other risk factors with distinctive predictive values. As a direction for the future study, prediction models with sensitivity and specificity higher than the ones reported in the present study should be sought in conjunction with data collected from multiple sites. In doing so, additional variables could be implemented in this model to establish a more comprehensive decision tree by which the holistic approach toward an individual with potential for developing diabetes could be precise and effective [32].

## 5. Conclusion

We proposed a classifier, based on a decision tree, to identify potential incidents of diabetes from a database of annual health check-up reports in a large Chinese hospital. We used nine features of patients which are easily obtained and noninvasive as predictor variables. The classifier, trained by J48, indicates that a decision tree analysis can be successfully applied to screen diabetes, which will support clinical practitioners for rapid diabetes identification. This type of work is essential in regions where the epidemiologic risk is high and medical expenses are unaffordable for most of the population. In addition, the proposed model provides a means to target the prevention of diabetes through community interventions, which could help improve early diabetes diagnosis and reduce the burden on the health system. Such study will be cost-effective when more chronic disease-related cares would be avoided through effective case management.

## Figures and Tables

**Table 1:** Characteristics of variables.

Variables	Possible values	Diabetes *N* = 1570	Nondiabetes *N* = 8866	*p* value
Age	18­34 years old	65 (4.1%)	5246 (59.2%)	<0.001
	35­49 years old	323 (20.6%)	2525 (28.5%)	
	50­65 years old	1182 (75.3%)	1095 (12.3%)	
Gender	Male	794 (50.6%)	2622 (29.6%)	<0.001
	Female	776 (49.4%)	6244 (70.4%)	
BMI	<25	540 (34.4%)	7322 (82.6%)	<0.001
	≥25	1030 (65.6%)	1544 (17.4%)	
Hypertension	Yes	462 (29.4%)	1572 (17.7%)	<0.001
	Nonhypertension	1108 (70.6%)	7294 (82.3%)	
Salty food preference	No	629 (40.1%)	7208 (81.3%)	<0.001
	Yes	941 (59.9%)	1658 (18.7%)	
History of cardiovascular disease or stroke	No	1311 (83.5%)	8169 (92.1%)	<0.001
	Yes	259 (16.5%)	697 (7.9%)	<0.001
Family history of diabetes	No	1038 (66.1%)	8092 (91.3%)	
	Yes	532 (33.9%)	774 (8.7%)	
Physical activity	Less	1299 (82.7%)	5043 (56.8%)	<0.001
	More	271 (17.3%)	3832 (43.2%)	
Work-related stress	Low	301 (19.2%)	2559 (28.9%)	0.002
	Moderate	804 (50.9%)	4950 (55.9%)	
	High	465 (29.9%)	1357 (15.2%)	

BMI: body mass index.

**Table 2:** The 19 rules extracted through the decision tree.

R1: IF age ≤ 49 and BMI < 25, THEN class: person is nondiabetic (4542)
R2: IF 34 < age ≤ 49 and BMI ≥ 25 and with a family history of diabetes, THEN class: person is diabetic (89)
R3: IF age ≤ 34 and BMI ≥ 25 and with a family history of diabetes, THEN class: person is nondiabetic (8)
R4: IF age ≤ 34 and BMI ≥ 25 and without a family history of diabetes, THEN class: person is nondiabetic (576)
R5: IF 34 < age ≤ 49 and BMI ≥ 25, without a family history of diabetes, with work-related stress, and without physical activity, THEN class: person is diabetic (21)
R6: IF 34 < age ≤ 49 and BMI ≥ 25, without a family history of diabetes, with work-related stress, and with physical activity, THEN class: person is nondiabetic (22)
R7: IF 34 < age ≤ 49 and BMI ≥ 25, without a family history of diabetes, without work-related stress, without hypertension, and without history of cardiovascular disease or stroke, THEN class: person is nondiabetic (330)
R8: IF 34 < age ≤ 49 and BMI > 25, without a family history of diabetes, without work-related stress, without hypertension, and with history of cardiovascular disease or stroke, THEN class: person is diabetic (18)
R9: IF 34 < age ≤ 49 and BMI ≥ 25, without a family history of diabetes, without work-related stress, with hypertension, and without physical activity, THEN class: person is diabetic (7)
R10: IF 34 < age ≤ 49 and BMI ≥ 25, without a family history of diabetes, without work-related stress, with hypertension, and with physical activity, THEN class: person is nondiabetic (19)
R11: IF age > 49 and BMI ≥ 25 and without work-related stress, THEN class: person is nondiabetic (59)
R12: IF age > 49 and BMI ≥ 25 and with work-related stress, THEN class: person is diabetic (517)
R13: IF age > 49 and BMI < 25 and with physical activity, THEN class: person is nondiabetic (275)
R14: IF age > 49 and BMI < 25, without physical activity, and without salty food preference, THEN class: person is nondiabetic (313)
R15: IF age > 49 and BMI < 25, without physical activity, with salty food preference, and with work-related stress, THEN class: person is diabetic (56)
R16: IF age > 49 and BMI < 25, without physical activity, with salty food preference, without work-related stress, and with hypertension, THEN class: person is diabetic (90)
R17: IF age > 49 and BMI < 25, without physical activity, with salty food preference, without work-related stress, without hypertension, and men, THEN class: person is diabetic (94)
R18: IF age > 49 and BMI < 25, without physical activity, with salty food preference, without work-related stress, without hypertension, women, and with history of cardiovascular disease or stroke, THEN class: person is diabetic (14)
R19: IF age > 49 and BMI < 25, without physical activity, with salty food preference, without work-related stress, without hypertension, women, and without history of cardiovascular disease or stroke, THEN class: person is nondiabetic (124)

R: abbreviation of rule.

**Table 3:** Confusion matrix of the test dataset.

Actual outcomes	Predicted outcome	
	Person without diabetes	Person with diabetes
Person without diabetes	2610	67
Person with diabetes	116	338

## Data Availability

The data used to support the findings of this study are available from the corresponding author upon request.

## Abbreviations

BMI: Body mass index
AUC: The area under the receiver operating characteristic curve
WHO: World Health Organization
SPSS: Statistical Package for the Social Sciences
WEKA: Waikato Environment for Knowledge Analysis.
